# A novel bone cement injector augments Chinese osteoporotic lumbar pedicle screw channel: a biomechanical investigation

**DOI:** 10.1186/s12891-022-05181-4

**Published:** 2022-04-12

**Authors:** Suochao Fu, Yu Zhang, Fuzhi Ai, Jianhua Wang, Zenghui Wu, Xiangyang Ma, Zixiang Wu, Zheng Wang, Wei Lei, Hong Xia

**Affiliations:** 1 Department of Orthopedics, General Hospital of Southern Theater Command of PLA, Guangzhou, 510000 People’s Republic of China; 2 Department of Orthopedics, Sun Yat-Sen Memorial Hospital, Sun Yat-Sen University, Guangzhou, 510020 People’s Republic of China; 3 Department of Spine Surgery, the Third Affiliated Hospital of Guangzhou Medical University, Guangzhou, 510000 People’s Republic of China; 4 Department of Orthopedics, the Fourth Medical Center of Chinese PLA General Hospital, Beijing, 100048 People’s Republic of China; 5 Fourth Department of Orthopedics, Xijing Hospital, Air Force Military Medical University, Xi’an, 733399 People’s Republic of China

**Keywords:** Pedicle injector, Lumbar pedicle screw, Osteoporosis, mechanical test, Human sample

## Abstract

**Background:**

The study aimed to (1) create a series of pedicle injectors with different number of holes on the sheath especially for the Chinese elderly patients and (2) further investigate the effects of the injectors on the augmentation of pedicle screw among osteoporotic lumbar pedicle channel.

**Methods:**

This study used the biomechanical test module of polyurethane (Pacific Research Laboratory Corp, USA) to simulate the mechanical properties of human osteoporotic cancellous bone. The bone cement injectors were invented based on anatomical parameters of lumbar pedicle in Chinese elderly patients. Mechanical test experiments were performed on the bone cement injectors according to the three groups, namely, a local augmentation group, a full-length augmentation group, and a control group. The local augmentation group included three subgroups including 4-hole group, 6-hole group, and 8-hole group. All holes were laterally placed. The full-length augmentation group was a straight-hole injector. The control group was defined that pedicle screws were inserted without any cement augmentation. Six screws were inserted in each group and the maximum insertion torque was recorded. After 24 h of injecting acrylic bone cement, routine X-ray and CT examinations were performed to evaluate the distribution of bone cement. The axial pull-out force of screws was tested with the help of the material testing system 858 (MTS-858) mechanical tester.

**Results:**

The bone cement injectors were consisted of the sheaths and the steel rods and the sheaths had different number of lateral holes. The control group had the lowest maximum insertion torque as compared with the 4-hole, 6-hole, 8-hole, and straight pore groups (*P* < 0.01), but the differences between the 4-hole, 6-hole, 8-hole, and straight pore groups were no statistical significance. The control group had the lowest maximum axial pull-out force as compared with the other four groups (*P* < 0.01). Subgroup analysis showed the 8-hole group (161.35 ± 27.17 N) had the lower maximum axial pull-out force as compared with the 4-hole group (217.29 ± 49.68 N), 6-hole group (228.39 ± 57.83 N), and straight pore group (237.55 ± 35.96 N) (*P* < 0.01). Bone cement was mainly distributed in 1/3 of the distal end of the screw among the 4-hole group, in the middle 1/3 and distal end of the screw among the 6-hole group, in the proximal 1/3 of the screw among the 8-hole group, and along the long axis of the whole screw body in the straight pore group. It might indicate that the 8-hole and straight-hole groups were more vulnerable to spinal canal cement leakage. After pullout, bone cement was also closely connected with the screw without any looseness or fragmentation.

**Conclusions:**

The bone cement injectors with different number of holes can be used to augment the pedicle screw channel. The pedicle screw augmented by the 4-hole or 6-hole sheath may have similar effects to the straight pore injector. However, the 8-hole injector may result in relatively lower pull-out strength and the straight pore injector has the risks of cement leakage as well as cement solidarization near the screw head.

## Introduction

Osteoporosis, a global health issue, is characterized by the disruption of the microarchitecture bone tissue and gradually reduced bone mass with aging [1–3]. The epidemiological prevalence of osteoporosis was 13% to 18% among the elderly in the United States [4], 21.2% in the Sweden [5], 23.5% in the France [1], and 15.7% in the People’s Republic of China [6]. With the advent of an aging society, the burden from osteoporosis and its relevant sequelae will continue to rapid increase [6, 7].

Osteoporosis patients were vulnerable to fractures including vertebral fracture, hip fracture, and distal forearm fracture mainly due to increased bone fragility. It was estimated that 1/2 female and 1/5 male aged above 50 years will developed an osteoporotic fracture during the period of their lifetime [8]. Vertebral osteoporotic compression fracture is the most common osteoporotic fracture. More explicitly, the incidence of vertebral fractures was about 20.0% globally among adults with an age of more than 50 years [9]. In China, it was estimated that the prevalence of vertebral osteoporotic compression fracture could be up to 1.11 million [10]. Vertebral osteoporotic compression fracture could lead to severe pain, dysfunction, and even disability. Besides, vertebral osteoporotic compression fracture was unpredictable and a substantial risk for additional fractures [11]. Osteoporosis and the subsequent fragility vertebral fractures had a significant impact on individual mortality, quality of life, morbidity, and social healthcare systems [12, 13].

Osteoporosis population suffering from vertebral fractures, which needed vertebrae internal fixation, usually encountered such an embarrassing condition that the bone was too fragile to have pedicle screw fixation [14, 15]. Pedicle screw fixation could be routinely achieved by the pedicle screw, which had been widely used in the treatment of spinal disorders [16]. Currently, it was characterized by 3-dimensional column fixation, convincing stabilization, and maintaining the reconstructed spinal alignment. However, pedicle screw fixation alone for osteoporosis population was not enough because screw loosening, pullout, and subsequent operation failure often occurs [14, 17]. Studies have shown that osteoporosis population had a significantly higher rate of screw loosening, as compared with normal bone mineral density patients [18]. Conventional pedicle screw could had a loosening rate of up to 62.8% [19]. Therefore, it was urgent and necessary to augment the stability of a pedicle screw fixation among osteoporosis patients.

This study aimed to invent a series of pedicle injectors and investigated the effects of the injectors with different number of holes on the augmentation of pedicle screw in the Chinese osteoporotic lumbar pedicle channel.

## Methods

### Study design

**Fig. 1:**
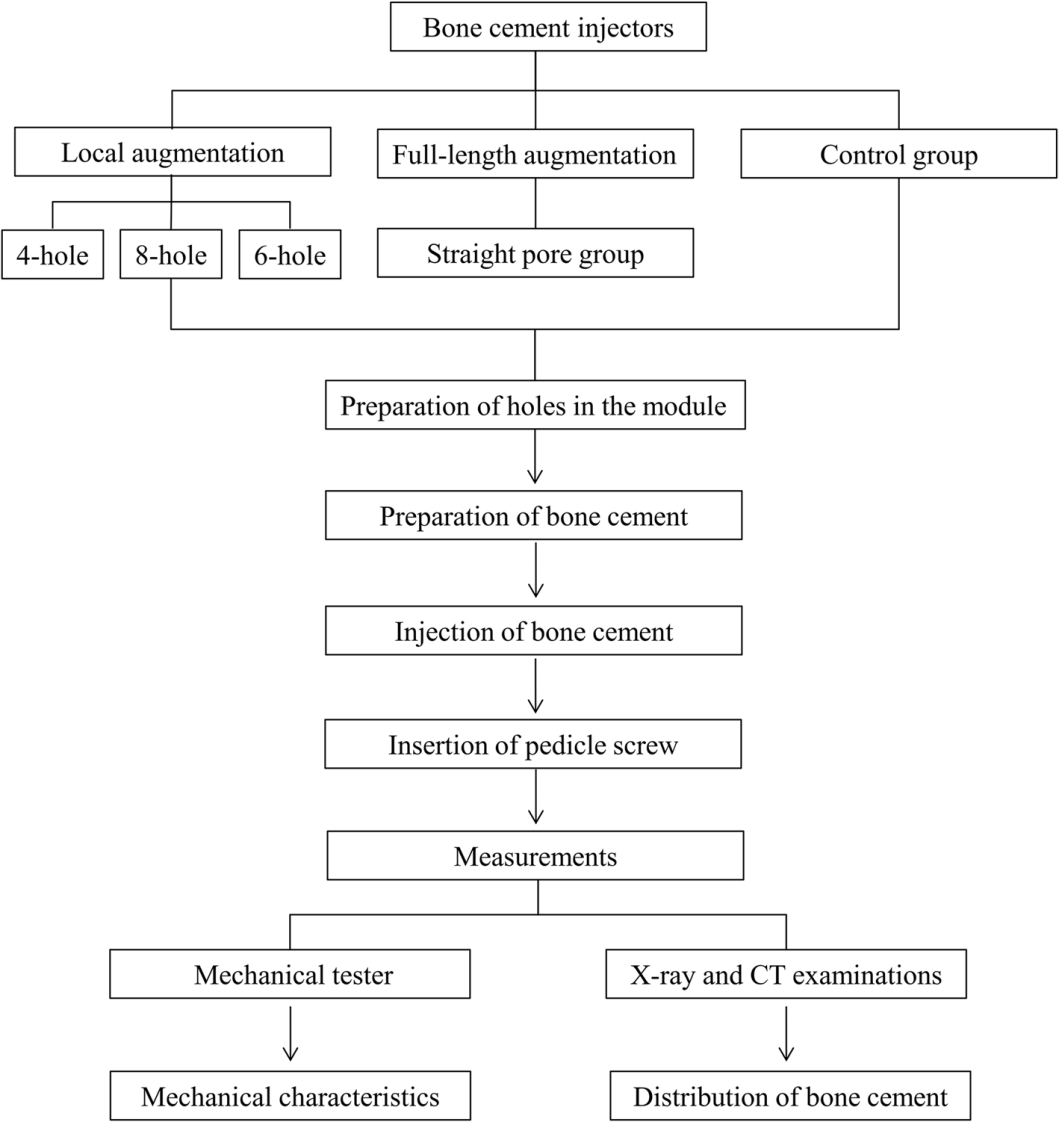
The flowchart of the study

The study used the biomechanical test module of polyurethane (Pacific Research Laboratory Corp, USA) to simulate the mechanical properties of human osteoporotic cancellous bone. The bone cement injectors were created based on anatomical parameters of lumbar pedicle among the Chinese. The target age group was about 50 years in the study. In the study, mechanical test experiments were performed based on the three groups, namely, a local augmentation group, a full-length augmentation group, and a control group. The local augmentation injectors had holes which were placed in lateral walls in the sheath. The local augmentation group included three subgroups including 4-hole group, 6-hole group, and 8-hole group. The full-length augmentation group included the injectors with a straight pore but without lateral holes. The study’s flowchart is shown in the Fig. 1. The Ethics Committee Board of the Air Force Military Medical University approved this study and this study was abided by the Declaration of Helsinki.

### Procedures

#### Preparation of holes in the module

A 3.5 mm diameter hand drill was used to prepare the pedicle screw channel in the biomechanical test module. A depth of 45 mm hole was drilled from the upper surface of the module. During the whole process, shaking of the hand drill was avoided, and accordingly artificial expansion of the hole was avoided in the cancellous module. All drilling operations were completed by the same doctor with rich clinical experience.

#### Preparation of bone cement

At room temperature, 2.50 g acrylic bone cement and 1.25 ml liquid were mixed together in a 50 ml steel cup, which was strictly accordance with the powder-liquid ratio of 2:1. Then, the bone cement was fully stirred with a stainless steel rod and sucked into the 5 ml syringe when the bone cement was paste. After inhalation, carefully removing the air between bone cement and the needle until the bone cement was toothpaste. After 2.50 g bone cement was prepared, the volume was about 2.5 ml, and the bone cement was ready for injection.

#### Injection of bone cement

In the local augmentation groups, the sheath was inserted along the prepared channel, and 2.50 ml of acrylic bone cement was injected into the sheath using a syringe. Then, after removing the syringe, a steel rod was putted into the hollow sheath to push out the remainder of the cement into the module entirely. In the full-length group, the sheath with a straight pore was inserted into the channel along the prepared hole in the module and then a syringe with acrylic bone cement was connected at the end of the sheath. The bone cement was injected into the module via the sheath and at the same time sheath was retrograded. When the bone cement was completed injected into the sheath, a steel rod was also putted into the sheath to push out the remainder of the cement into the module and meanwhile the sheath continued to retrograded until the sheath was out of the prepared hole in the module.

#### Insertion of pedicle screw

**Fig. 2:**
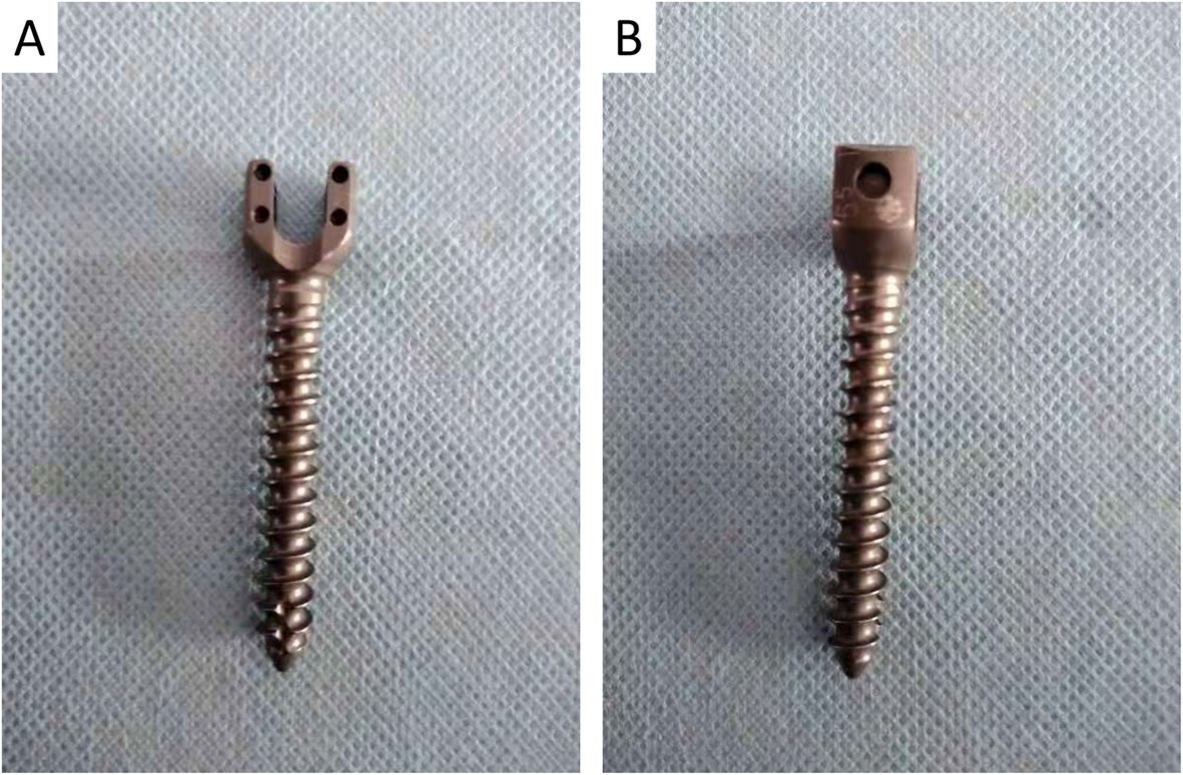
The general appearance pedicle screw used in the study. **A** The front view of the pedicle screw; **B** The lateral view of the pedicle screw

**Fig. 3:**
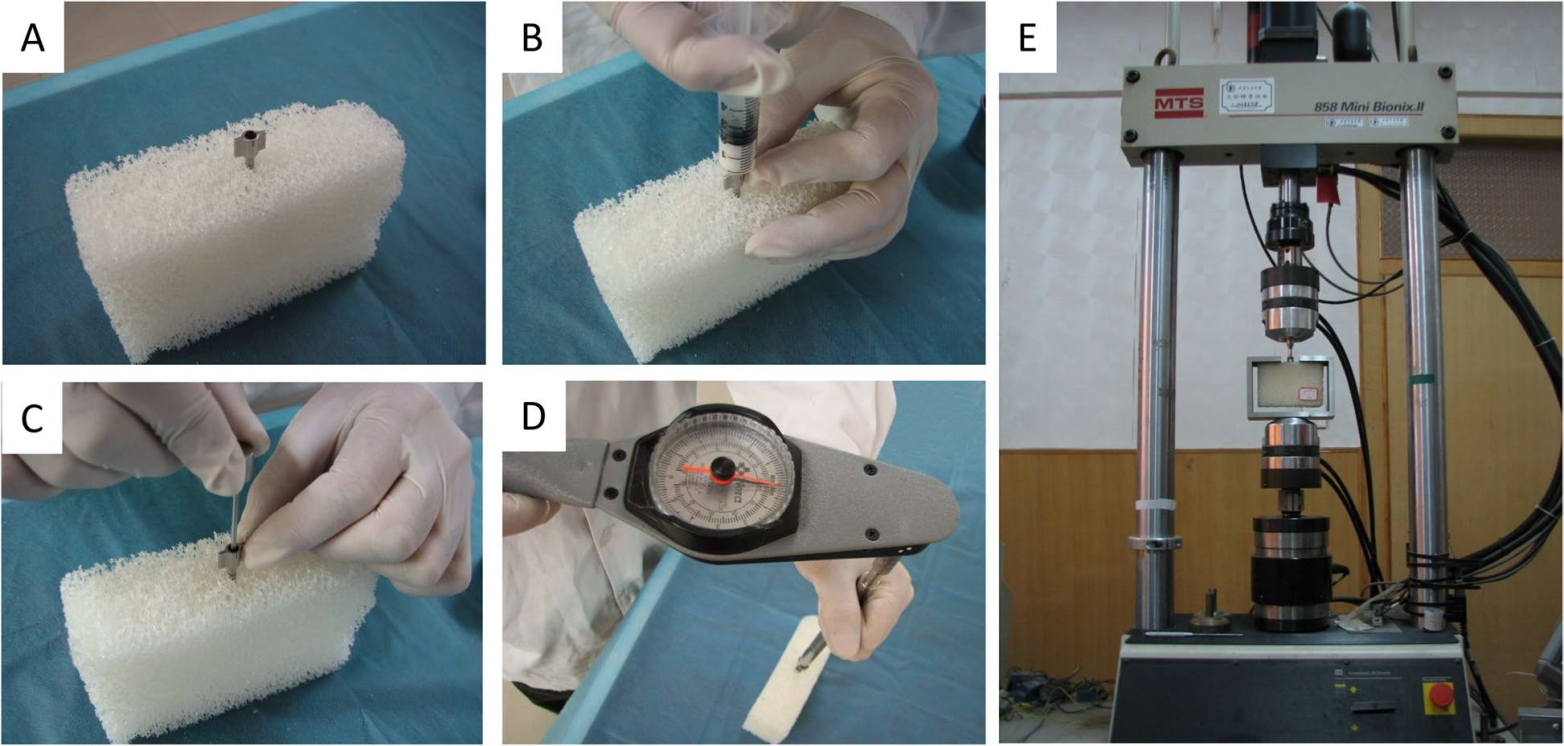
Injection of bone cement, insertion of pedicle screw and a mechanical tester. **A** The sheath of the bone cement injector was inserted along the prepared channel in the biomechanical test module of polyurethane (The white cuboid block); **B** 2.5 ml of acrylic bone cement mixed with 1.25 ml liquid and was injected into the sheath; **C** A steel rod was putted into the hollow sheath to push out the remainder of the acrylic bone cement into the module; **D** The CD HORIZON M8 pedicle screw was inserted into the channel with a manual torque wrench at a rate of 3 rev/min evenly by a torque wrench; **E** The MTS-858 mechanical tester tested the mechanical characteristics of the module

Before the cement was hardened, the CD HORIZON M8 pedicle screw (size: length 45 mm and diameter 6.5 mm, Sofamor Danek Corp USA, Fig. 2) was inserted into the channel with a manual torque wrench at a rate of 3 rev/min evenly by a torque wrench (WERA company, German) according to the criteria of American Society for Testing and Materials (ASTM) F543-02 (Fig. 3). The control group was defined that pedicle screws were inserted without any cement augmentation. Six screws were inserted in each group.

### Measurements

When screws were inserted, the maximum torque was recorded. After 24 h of injecting acrylic bone cement, routine X-ray and CT examinations were performed to evaluate the distribution of bone cement. The axial pull-out force of screws was tested using the material testing system 858 (MTS-858) mechanical tester (Fig. 3E). We strictly ensured that the rod of the pedicle screw and the upper and lower clamps were on the same axis. Applying 2 N preload until the test module was in close contact with the module, and then the load was cleared. The pedicle screw was pulled out at a speed of 5 mm/min in accordance with ASTM F 543–02. The maximum axial pull-out force of the screw was defined as the highest point of the screw pull-out loading displacement curve when the module was damaged.

#### Statistical analysis

Quantitative data, such as the maximum insertion torque and maximum axial pull-out force, were presented as mean ± standard deviation (SD). Analysis of variance, supplied by SNK-q test, was used to comparison between groups. P-value of less than 0.05 indicates statistical significance. All data were analyzed by the SPSS software (IBM Corp, version 11.5, Armonk, New York).

## Results

### Creation of novel bone cement injectors

**Fig. 4:**
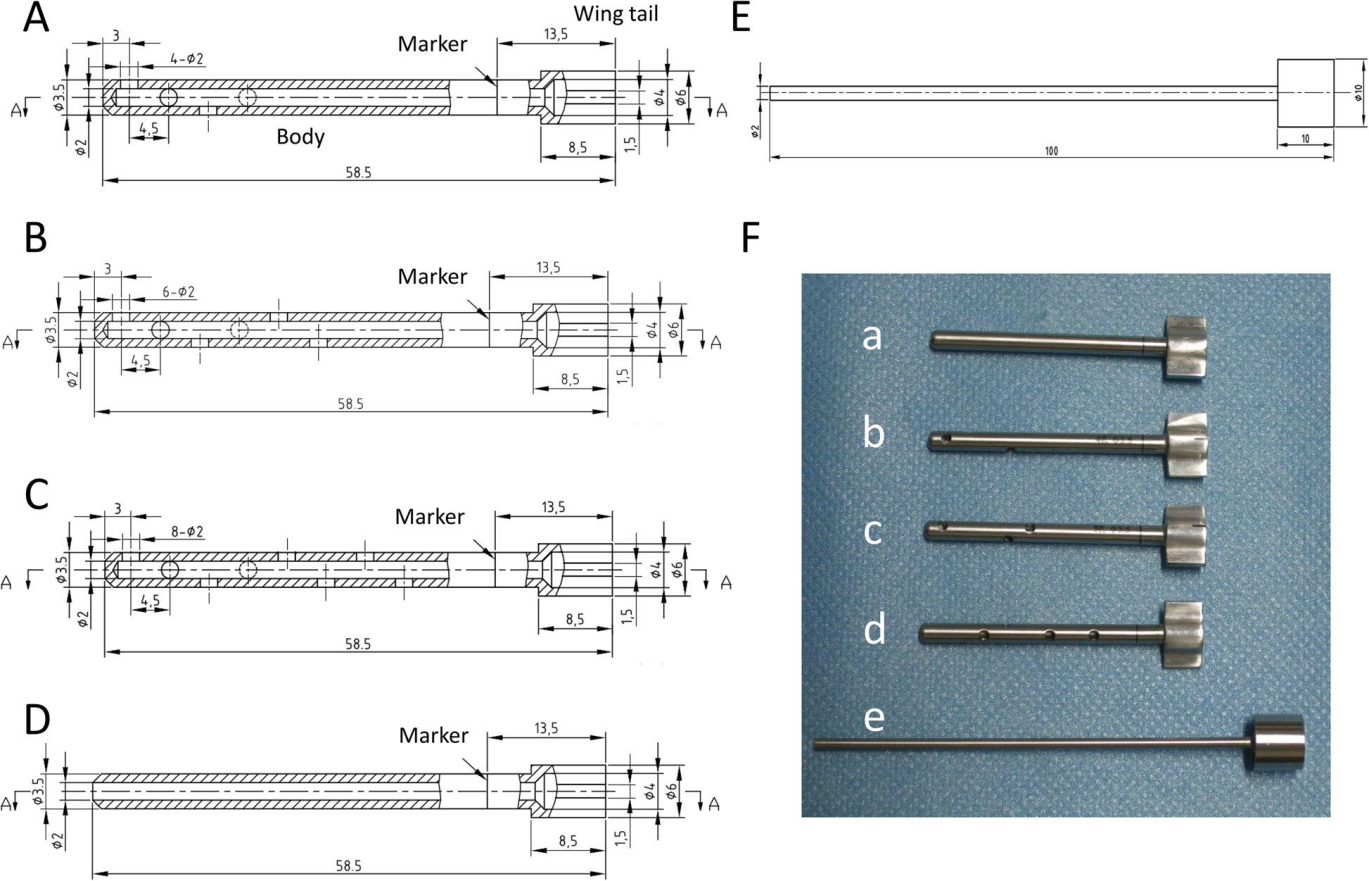
Design and parameters of bone cement injectors. **A** The 4-hole sheath; **B** The 6-hole sheath; **C** The 8-hole sheath; **D** The straight pore sheath; **E** The steel rod for the sheath; **F** The physical looking of the injectors (a indicates the straight pore sheath, b indicates the 4-hole sheath, c indicates the 6-hole sheath, d indicates the 8-hole sheath, and e indicates the steel rod)

The bone cement injectors were invented based on the anatomical parameters of lumbar pedicle in the Chinese. The bone cement injectors were consisted of the sheaths and the steel rods. The sheaths had a wing tail and a body, and the parameters of sheaths are shown in Fig. 4. The inner diameter of the wing tail was 4 mm, which could be used to connect with a 5 ml medical syringe. The sheaths were hollow inside with an inner diameter of 2.0 mm and an outer diameter of 3.5 mm. A marked line was placed at the 45.0 mm from the distal end, which represented the direction of 12 o'clock. The sheaths had different number of lateral holes, including 4 lateral holes (Fig. 4A), 6 lateral holes (Fig. 4B), and 8 lateral holes (Fig. 4C), and the sheaths with a straight pore but without any lateral holes (Fig. 4D). The lateral holes were 2 mm diameter round hole. According to the direction of looking from the wing tail of the sheath to the front section of the sheath, the first hole was located at the 12 o'clock position at the farthest end, the second hole was located at the 3 o'clock direction close to the wing tail, and the distance from the center of the first hole is 4.5 mm. Accordingly, each additional hole was horizontally close to the proximal end of the wing tail by 4.5 mm, i.e. the third hole was located at the 6 o'clock direction, and the fourth hole was located at the 9 o'clock direction. Finally, the 8th hole was located at the 6 o'clock direction at the proximal end of the wing tail. Figure 4E shows the steel rod for the sheath. The physical looking of all the injectors is shown in Fig. 4F.

### The maximum insertion torque and axial pull-out force

**Fig. 5:**
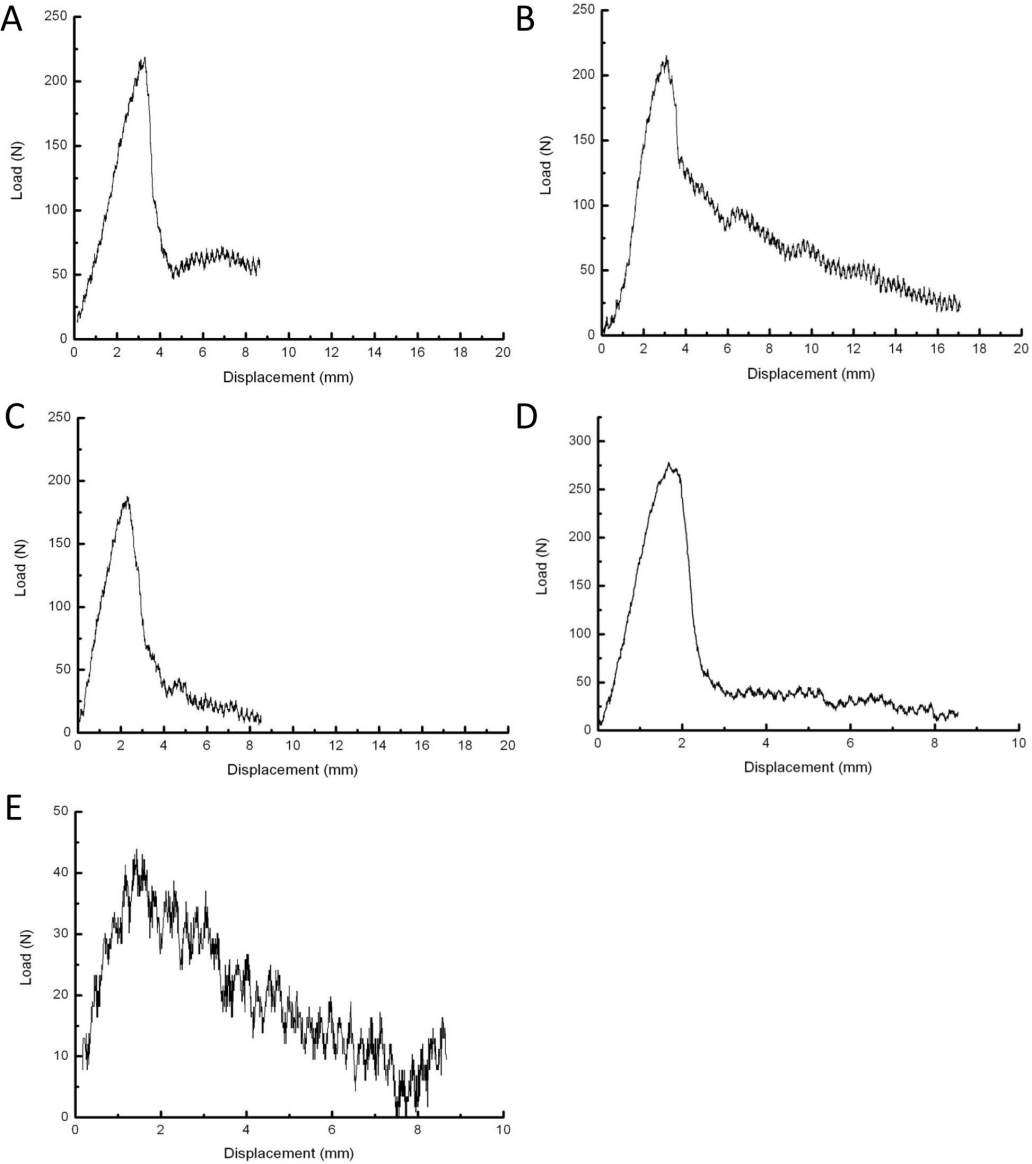
Loading displacement curves. **A** The 4-hole group; **B** The 6-hole group; **C** The 8-hole group; **D** The straight pore group; **E** The control group

The control group had the lowest maximum insertion torque as compared with the 4-hole, 6-hole, 8-hole, and straight pore groups (P < 0.01), but the difference between the 4-hole, 6-hole, 8-hole, and straight pore groups was no statistical significance (Table 1). The control group had the lowest maximum axial pull-out force as compared with the other four groups (*P* < 0.01). The SNK-q test also showed that the 8-hole group had the lower maximum axial pull-out force as compared with the 4-hole, 6-hole, and straight pore groups and the difference was statistical significance (*P* < 0.01). Fig. 5 shows the loading displacement curves in each group.

**Table 1:** The maximum insertion torque and axial pull-out force

Parameters	Number of lateral holes					*P* ^1^
	4 holes	6 holes	8 holes	straight pore	control	
Maximum insertion torque (N·m)	0.12 ± 0.01	0.12 ± 0.02	0.11 ± 0.01	0.11 ± 0.01	0.07 ± 0.01^2^	< 0.01
Maximum axial pull-out strength (N)	217.29 ± 49.68	228.39 ± 57.83	161.35 ± 27.17^3^	237.55 ± 35.96	40.37 ± 8.9^4^	< 0.01

*Note:* 1 indicates the *P*-value was obtained from the analysis of variance; 2 indicates statistical significance as compared to the other four groups according to the SNK-q test; 3 indicates statistical significance as compared to the other four groups according to the SNK-q test; 4 indicates statistical significance as compared to the other four groups according to the SNK-q test

### The distribution of bone cement in module

**Fig. 6:**
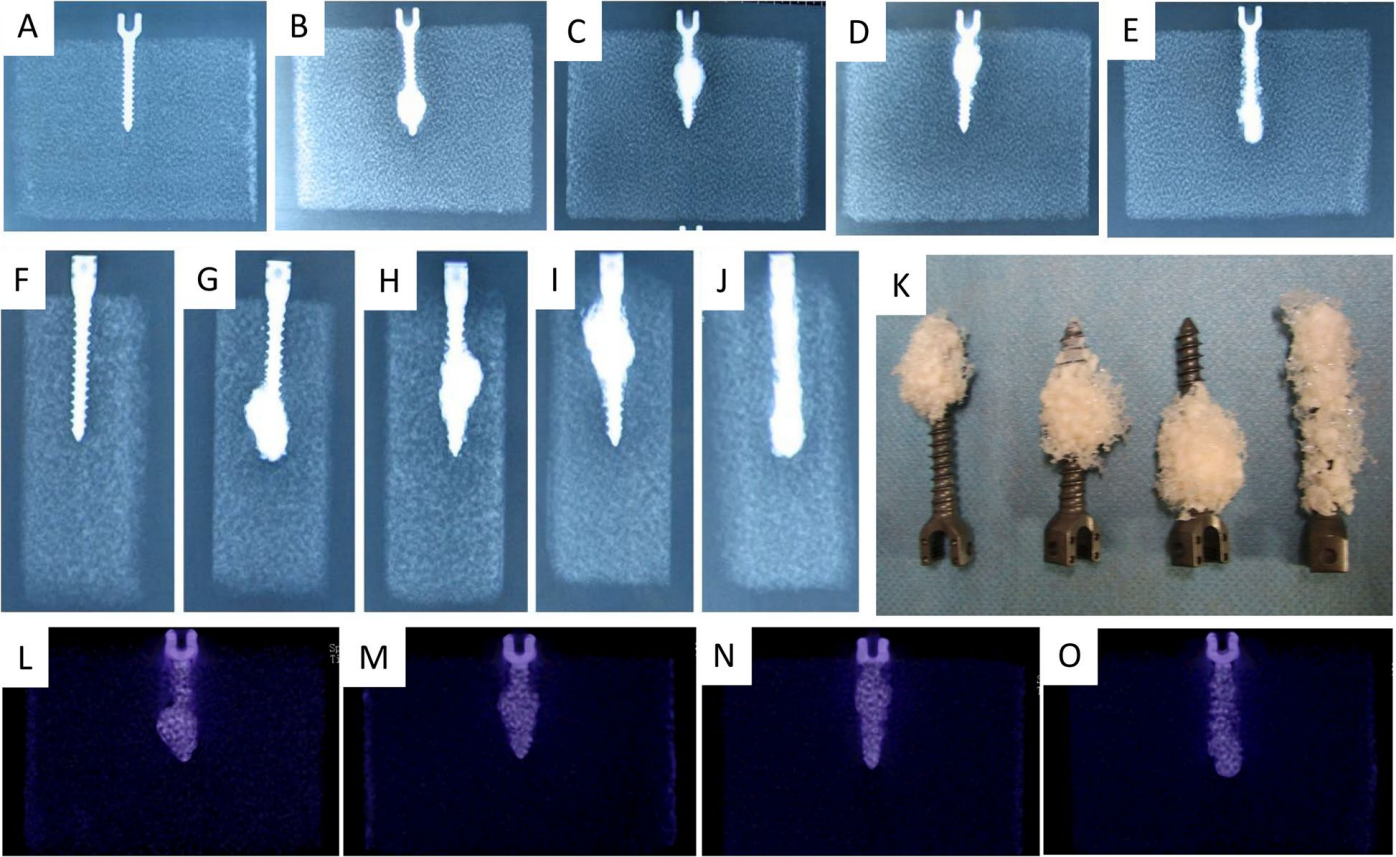
The distribution of bone cement in module based on X-ray and CT scan. **A** Anterior posterior view of the control group; **B** Anterior posterior view of the 4-hole group; **C** Anterior posterior view of the 6-hole group; **D** Anterior posterior view of the 8-hole group; **E** Anterior posterior view of the straight pore group; **F** Lateral view of the control group; **G** Lateral view of the 4-hole group; **H** Lateral view of the 6-hole group; **I** Lateral view of the 8-hole group; **J** Lateral view of the straight pore group; **K** Physical looking after pulling out (the first was the 4-hole group, the second was the 6-hole group, the third was the 8-hole group, and the fourth was the straight pore group); **L** CT scan of the 4-hole group; **M** CT scan of the 6-hole group; **N** CT scan of the 8-hole group; **O** CT scan of the straight pore group

Acrylic bone cement was not injected in the control group (Fig. 6A). It was mainly distributed in 1/3 of the distal end of the screw in the 4-hole group (Fig. 6B), showing a spiral trend. Acrylic bone cement was mainly distributed in the middle 1/3 and distal end of the screw among the 6-hole group (Fig. 6C), in the proximal 1/3 of the screw among the 8-hole group (Fig. 6D), and along the long axis of the whole screw body in the straight pore group (Fig. 6E). In the study, 6-hole group and 8-hole group also showed a spiral trend. Figure 6F to 6J shows lateral view of the pedicle screw in the module. It found that the interface between the screw and bone cement was firmly stick together (Fig. 6K), and the bone cement was still tightly wrapped with it when pulling it out using the MTS-858 mechanical tester. We also found that when bone cement was injected with an 8-hole injector, there was an overflow phenomenon of bone cement leaking into to the root of the screw head (Fig. 6K). It was also easy to find the overflow at the beginning of the channel in the straight-hole group (Fig. 6K). Similar distribution of bone cement in module was also observed based on CT scan (Fig. 6L to 6O). The above-mentioned results indicated that the 8-hole and straight-hole group might be more vulnerable to spinal canal cement leakage, as compared with the 4-hole and 6-hole groups. Bone cement was closely connected with the screw without any looseness or fragmentation. There is no gap and crack between bone cement and polyurethane on the fracture surface, indicating that bone cement had good mechanical properties.

## Discussion

This study created a series of novel bone cement injectors and further investigated the effects of the injectors with different number of holes on the augmentation of pedicle screw in osteoporotic lumbar pedicle channel. Maximum insertion torque, maximum axial pull-out strength, and the distribution of bone cement in module were evaluated in the study among groups with different number of holes in the injector’s sheaths. This study showed that the bone cement injectors with different number of holes might have different characteristics.

Several studies have reported approaches to improve the stability of pedicle screws, including enlarging the diameter of pedicle screws [20], modifying the screw thread [21], performing injectable pedicle screw [22], proper pedicle screw selection [23], fused lumbar spine [24], and directly injecting bone cement into the pilot hole [25]. A biomechanical study had proved that solid screws with retrograde cement pre-filling was capable of achieving improved initial fixation strength as compared to cannulated screws with cement injection via perforation [26]. Our study aimed to create a new series of bone cement injectors which were designed to guide the distribution of bone cement so that bone cement pre-filling could be controlled. To our knowledge, this study was the first to invest a series of bone cement injectors with different sheath holes and compare their effects on screw augmentation.

This study found that the 4-hole group, 6-hole group, 8-hole group, and straight pore group had similar maximum insertion torque. Namely, there was no significant difference in the maximum insertion torque of the screw after the screw channel was strengthened by the bone cement with different number of holes. The torque values increased significantly from 0.07 N·m in control group to about 0.12 N·m in bone cement hole groups. It suggested that this phenomenon was closely relevant to the filling of bone cement surrounding the material in the module after bone cement injection. Bone cement filled the surrounding loose structure to form a locally dense structure. Compared with the loose porous structure, the contact area increased when screws were inserted in, thus the friction resistance increased accordingly, which was shown as the increase of torque.

This study further observed that the 8-hole group had lower maximum axial pull-out strength as compared with the 4-hole, 6-hole, and straight-hole groups. Theoretically, after the screw was inserted in the module, the main factors affecting the maximum pull-out strength were the firmness of the interface between the material and the nail and the shear strength of the surrounding materials. According to the theory of solid mechanics, after bone cement formed a solid wrapped mass around the screw, it is necessary to overcome not only the shear force due to the surrounding material, but also the resistance of the material along the pulling out process between the wrapped mass and the screw head after micro-fracture around the wrapped mass. When bone cement which was injected into the module would form a close wrapped mass with the metal screw, which was equivalent to increasing the diameter of the screw. The control group did not receive cement injection, thus the control group had the lowest maximum axial pull-out strength. The 8-hole group had the second lowest maximum axial pull-out strength mainly because bone cement was morphologically distributed in the proximal 1/3 of the screw so the resistance strength was limited. Regarding the straight-hole group, bone cement was distributed along the axis of the whole screw, so it had the biggest contact with the material, which contributed to large resistance strength. And there was no significant difference in pullout strength between the 4-hole group and 6-hole group under osteoporotic condition despite the fact that the two groups had different bone cement distributions. Polymethyl methacrylate (PMMA) could provide 213% axial resistance strength, acrylic bone cement contained PMMA and it showed higher axial resistance strength as compared with PMMA. The 8-hole group also increased from 40.37 ± 8.94 N (the control group) to 161.35 ± 27.17 N, which indicated a 400% improvement of maximum axial pull-out strength (N).

We found that bone cement was regularly and morphologically distributed in the module. In the 4-hole group, bone cement was mainly distributed in 1/3 of the distal end of the screw. Bone cement was mainly distributed in the middle 1/3 and distal end of the screw in the 6-hole group, in the proximal 1/3 of the screw in the 8-hole group, and along the long axis of the whole screw body in the straight pore group. The biomechanical module used in our study was polyurethane, which has the characteristics of uniform material and bone mineral density, which was capable of reducing the influence of bone mineral density on the pullout force of pedicle screws. As a commercial material, it has been internationally recognized and widely used [27–29]. We found that when bone cement was injected using an 8-hole injector, there was an overflow phenomenon of bone cement leaking into to the root of the screw head. Besides, we also observed that the overflow was presented at the beginning of the channel in the straight-hole group. The above-mentioned results indicated that the 8-hole and straight-hole group might be more vulnerable to spinal canal cement leakage, as compared with the 4-hole group and the 6-hole group. According to the morphological distribution wrapped around the screw after cement injection, we believed that bone cement in the 8-hole group was almost distributed near the head of the pedicle screw, and it was relative easy for bone cement to overflow into the vertebral canal when the channel was close to the vertebral canal. The straight-hole group had the largest maximum axial pull-out strength, which might result in difficulty in surgical revision.

In a word, the 8-hole group had the lowest maximum axial pull-out strength except for the control group and the 8-hole group might be more vulnerable to spinal canal cement leakage. The straight-hole group had the largest maximum axial pull-out strength and this group might be also more vulnerable to spinal canal cement leakage. Besides, the high maximum axial pull-out strength might result in difficulty in surgical revision. Consequently, these results indicated that the 8-hole group and the straight-hole group both might not be appropriate to be used as a bone cement injector. The 4-hole group and 6-hole sheath groups had similar and satisfactory maximum axial pull-out strength except and the both were not vulnerable to spinal canal cement leakage, all of which indicated that the both group were more appropriate to be used as a bone cement injector.

### Limitations

The study had several limitations. Firstly, the indications for the use of the bone cement injector in osteoporosis were not clearly defined since we still lacked support from evidence-based medicine. Secondly, the appropriate bone cement volumes for vertebrae were disputable since different levels of vertebrae might have different volumes of bone cement to achieve an absolute stable fixation. Thirdly, the biomechanical tests were only conducted at screw implantation after a limited cyclic loading cycle, but human body is a whole and cyclic loading always exists, so the results might not be applicable totally. Lastly, we only tested the injectors in the osteoporotic cancellous bone and, in fact, a whole bone also included cortical bone, and it is difficult to accurately model and simulate needle placement in spinal surgery using the geometry and material properties of the modeling vertebra [3]. Thus, further investigations are still needed in the future.

## Conclusions

The bone cement injectors with different number of holes can be used to augment the pedicle screw channel. The pedicle screw augmented by the 4-hole or 6-hole sheath may have similar effects to the straight pore injector. However, the 8-hole injector may result in relatively lower pull-out strength and has the possibility of cement leakage as well as cement solidarization near the screw head.

## Data Availability

The data are available upon reasonable request to the corresponding author.
